# Association between coronavirus disease 2019 and new-onset autoimmune diseases during the early phase of the pandemic

**DOI:** 10.1371/journal.pone.0347872

**Published:** 2026-05-05

**Authors:** Joung Ha Park, Min-Taek Lee, Sun-Young Jung, Sang Tae Choi, Seong-Ho Choi

**Affiliations:** 1 Division of Infectious Diseases, Department of Internal Medicine, Chung-Ang University Gwangmyeong Hospital, Gwangmyeong-si, South Korea; 2 College of Pharmacy, Chung-Ang University, Seoul, South Korea; 3 Department of Global Innovative Drugs, The Graduate School of Chung-Ang University, Seoul, South Korea; 4 Division of Rheumatology, Department of Internal Medicine, Chung-Ang University College of Medicine, Seoul, South Korea; 5 Division of Infectious Diseases, Department of Internal Medicine, Chung-Ang University Hospital, Seoul, South Korea; 6 Biomedical Research Institute, Chung-Ang University Hospital, Seoul, South Korea; 7 Department of Internal Medicine, Naeun Hospital, Seo-gu, Incheon, South Korea; Independent Medical Researcher and Writer, UNITED KINGDOM OF GREAT BRITAIN AND NORTHERN IRELAND

## Abstract

Emerging evidence suggests that viral infections, including SARS-CoV-2, may trigger autoimmunity. This study investigated the risk of developing autoimmune diseases following coronavirus disease 2019 (COVID-19) using sequence symmetry analysis. We utilized nationwide population-based data from South Korea by linking the National Health Insurance Service database with the Korea Centers for Disease Control and Prevention Agency COVID-19 registry. This study included 2,678 patients with COVID-19 and 92,725 patients without COVID-19 identified between 01/10/2020 and 30/06/2021, during the early phase of the pandemic. Both groups consisted of individuals who were diagnosed with autoimmune diseases within 180 days before or after the index date. We calculated the adjusted sequence ratio (aSR) to compare the incidence of autoimmune diseases within 180 days before and from 14 to 180 days after the index date (the COVID-19 diagnosis date for the COVID-19 group and the first medical visit for the non-COVID-19 group). The differences in autoimmune disease incidence between the groups were evaluated using the ratio of aSR (RaSR). The incidence of newly diagnosed autoimmune diseases—Behcet’s disease (RaSR, 2.03), ankylosing spondylitis (2.04), ulcerative colitis (1.15), Crohn’s disease (2.22), psoriasis (1.27), type 1 diabetes mellitus (1.61), and Graves’ disease (1.14)—was significantly higher in the COVID-19 group than in the non-COVID-19 group after the index date. Subgroup analysis comparing patients with non-severe COVID-19 with those without COVID-19 yielded consistent findings. Furthermore, the incidence of inflammatory bowel diseases was higher in the non-severe COVID-19 group after the index date (RaSR, 1.29). These findings reinforce growing evidence that COVID-19 could induce autoimmunity and increase the risk of developing autoimmune diseases. Therefore, clinicians should remain vigilant for potential autoimmune complications in patients with a history of COVID-19 when clinically indicated.

## Introduction

The coronavirus disease 2019 (COVID-19), caused by severe acute respiratory syndrome coronavirus 2 (SARS-CoV-2), led to a global pandemic from 2020 to 2023 and transitioned into an endemic phase in May 2023. SARS-CoV-2 enters human cells via the angiotensin-converting enzyme 2 (ACE2) receptor, causing direct cellular injury and dysregulated immune and inflammatory responses. Through these mechanisms, the virus affects not only the lungs–causing pneumonia and acute respiratory distress syndrome (ARDS) – but also multiorgan systems.

Viral infections have long been recognized as environmental triggers for autoimmune diseases [1,2]. The COVID-19 pandemic provides a unique opportunity to explore and deepen our understanding of the association between viral infections and autoimmunity. Several cases of new-onset autoimmune diseases following COVID-19 have been reported [3–8]. In particular, SARS-CoV-2 has been suggested to trigger autoimmune diseases through mechanisms such as molecular mimicry, bystander activation, epitope spreading, and dysregulated cytokine release, all of which are associated with aberrant immune responses [1,2,9,10]. Nevertheless, it remains uncertain whether the pandemic has led to a true increase in the incidence of autoimmune diseases. This raised an important clinical question: should autoimmune disease be considered as one of the potential long-term complications that require careful post-COVID-19 monitoring and management?

Several studies have examined the occurrence of new-onset autoimmune disease following COVID-19 [3–8]. However, these studies compared the incidence of autoimmune diseases between individuals with COVID-19 and age- and sex-matched controls without a history of infection. Given that COVID-19 may have influenced hospital accessibility and healthcare-seeking behaviors, especially during the pandemic period, such comparisons may be subject to selection and ascertainment biases. To mitigate these limitations, this study employed sequence symmetry analysis (SSA) using nationwide population-based data to assess asymmetries in the ratio of temporal sequences of autoimmune disease incidence between patients with and without COVID-19.

## Materials and methods

### Study design and setting

This study was a nationwide population-based retrospective explanatory cohort study and followed the Reporting of studies Conducting using Observational Routinely collected health Data (RECORD) guidelines for reporting observational studies using routinely collected health data [11]. This study utilized the K-COV-N cohort (Korea Disease Control and Prevention Agency-COVID-19-National Health Insurance Service cohort) database (KDCA-NHIS-2023-1-091), which is the nationwide population-based data, between 01/10/2020 and 30/06/2021.

### Data sources and measurements

The NHIS, which is a single insurer, covers more than 98% of the population in South Korea. The NHIS database includes general demographic information, healthcare utilization data, diagnostic codes, and prescription data from all healthcare institutions nationwide. This database is linked to KDCA COVID-19 registry, which contains details such as the birth date, sex, and date of confirmed SARS-CoV-2 infection. Diagnoses were coded according to the International Classification of Diseases, 10th Revision (S1 Table). The baseline characteristics included age, sex, and underlying disease, which were defined based on the diagnoses recorded within 1 year prior to the index date. Underlying diseases included myocardial infarction, congestive heart failure, peripheral vascular disease, cerebrovascular disease, dementia, chronic pulmonary disease, peptic ulcer disease, mild liver disease, moderate or severe liver disease, diabetes mellitus (DM) with complications, DM without complications, hemiplegia or paraplegia, renal disease, malignancy, metastatic solid tumor, and human immunodeficiency virus/acquired immunodeficiency syndrome. The investigators had access only to de-identified data extracted by the NHIS and were responsible for defining and selecting the study population.

### Participants

We included individuals who were either diagnosed with SARS-CoV-2 infection or visited a clinic for other causes between 01/10/2020 and 30/06/2021. During the study period, all cases of COVID-19 confirmed by real-time polymerase chain reaction testing were mandatorily reported to the national registry in South Korea. Thus, individuals without a record of confirmed COVID-19 were classified as the non-COVID-19 group. The index date was defined as the date of COVID-19 diagnosis for the COVID-19 group and the date of the first medical visit for the non-COVID-19 group. We evaluated the association between COVID-19 and new-onset autoimmune diseases using SSA. Conceptually, the SSA method is used to investigate asymmetries in the incidence of an event occurring before and after the index date within a fixed observation period. We defined the fixed observation period as 180 days before and from 14 to 180 days after the index date (exposure window). In the SSA, the exposure window was defined based on the expected time to onset of the outcome following COVID-19 infection. A 14-day lag period was applied to account for the potential induction period of autoimmune conditions and to minimize protopathic bias [12]; therefore, autoimmune diseases occurring within 14 days after the index date were not considered events. Finally, we included individuals who had at least one diagnosis of autoimmune disease within either 180 days before or from 14 to 180 days after the index date in both COVID-19 and non-COVID-19 groups. Individuals with a prior diagnosis of the same autoimmune disease within one year before the first diagnosis during the SSA period were excluded from the analysis.

### Exposure and outcomes

The exposure of interest was SARS-CoV-2 infection, which was identified through the KCDA COVID-19 registry. Severe COVID-19 was defined as cases with a documented history of intensive care unit (ICU) admission, mechanical ventilation, conventional oxygen therapy or high-flow nasal cannula (HFNC), or extracorporeal membrane oxygenation (ECMO). Individuals without any registry record of confirmed infection between 01/10/2020 and 30/06/2021 were classified as the non-COVID-19 group.

In this study, the outcome was defined as the temporal asymmetry in new-onset autoimmune disease diagnoses before and after the index date. A new-onset autoimmune disease was defined as a diagnosis recorded on at least two separate occasions during the SSA period. Autoimmune diseases included systemic lupus erythematosus (SLE), systemic sclerosis (SSc), idiopathic inflammatory myopathy (IIM), Sjögren disease (SjD), mixed connective tissue disease (MCTD), Behcet’s disease (BD), polymyalgia rheumatica (PMR), rheumatoid arthritis (RA), ankylosing spondylitis (AS), adult-onset Still’s disease (AOSD), Crohn’s disease (CD), ulcerative colitis (UC), autoimmune hepatitis (AIH), granulomatosis with polyangiitis (GPA), microscopic polyangiitis (MPA), eosinophilic granulomatosis with polyangiitis (EGPA), polyarteritis nodosa (PAN), Takayasu’s arteritis (TA), multiple sclerosis (MS), psoriasis, type 1 DM, Hashimoto’s thyroiditis, and Graves’ disease (S1 Table). We categorized autoimmune diseases into three groups: autoimmune rheumatic diseases, inflammatory bowel diseases, and autoimmune endocrine diseases. Autoimmune rheumatic diseases included SLE, SSc, IIM, SjD, MCTD, BD, PMR, AOSD, GPA, MPA, EGPA, PAN, TA, RA, and AS. Inflammatory bowel diseases included UC and CD. Autoimmune endocrine diseases included type 1 DM, Graves’ disease, and Hashimoto’s disease. We included certain autoimmune-autoinflammatory diseases (e.g., inflammatory bowel disease and psoriasis) [13,14], as these diseases share immune-mediated pathogenic mechanisms with classical autoimmune diseases and are commonly included in ICD-based epidemiologic studies [3–8].

### Statistical methods

We collected baseline characteristics, including age, sex, and underlying diseases, of patients with and without COVID-19 and conducted SSA to identify asymmetries in autoimmune disease incidence before and after COVID-19 diagnosis [15,16]. SSA was originally developed as a post-marketing surveillance for newly marketed medications [17,18]. However, we selected SSA for this study because of its methodological advantages over traditional cohort-based statistical approaches, particularly its ability to inherently adjust for time-invariant confounders. The primary measure of risk in SSA was the sequence ratio (SR), which was calculated by dividing the number of cases of autoimmune diseases occurring after COVID-19 diagnosis by those occurring before the diagnosis. The crude SR represents the ratio of post-exposure to pre-exposure incidence, providing an estimate of the temporal association. However, the SR can be biased by underlying temporal trends in disease incidence and time-varying confounders. To adjust for this, we calculated the aSR by dividing the crude SR by the null-effect SR (neSR): aSR = crude SR/neSR. If a temporal association is absent, the aSR would be equal to 1. The neSR accounts for background temporal trends by estimating the expected SR assuming no causal association. It was calculated as follows:


$$Null-effectSR=\frac{P_{a}}{\text{1}-P_{a}}$$

where P_*a*_ represents the overall probability of autoimmune disease occurrence after the index date, derived from incidence patterns in the background population [16,18,19].


$$P_{a}=\frac{\sum_{m=\text{1}}^{\mu }\left[I_{m}*(\sum_{n=m+\text{1}}^{m+d}M_{n})\right]}{\sum_{m=\text{1}}^{\mu }[I_{m}*((\sum_{n=m-d}^{m-\text{1}}M_{n})+(\sum_{n=m\_\text{1}}^{m+\text{1}}M_{n}))]}$$

where *μ* indicates the last day of the study period, *m* indicates the consecutive day of SARS-CoV-2 infection during the study, *I*_*m*_ is the number of persons with SARS-CoV-2 infection on a specific day, *d* indicates the length (in days) of the time interval to capture event occurrence, *n* indicates the consecutive days of the study period, and *M*_*n*_ is the number of patients with an autoimmune disease on a specific day. P_*a*_ reflects background temporal trends in autoimmune disease incidence and represents the expected asymmetry between events occurring before and after the index date under the null hypothesis of no causal association. The neSR is calculated using P_*a*_ to adjust the crude SR for these underlying temporal trends. Autoimmune disease incidence was determined within the time interval on a given day. The time interval was defined as 28 days based on previous study applying SSA [20]. As the appropriate length of time interval may vary depending on the characteristics of the exposure and outcome, a sensitivity analysis using a 14-day interval was additionally performed. Accordingly, the incidence of autoimmune diseases was assessed with a 28-day interval within 180 days before and from 14 to 180 days after index date. The difference in the asymmetry of autoimmune disease occurrence between the COVID-19 and non-COVID-19 groups was evaluated using the ratio of aSRs (RaSRs). The RaSR reflects the relative difference in temporal asymmetry of event sequences between groups rather than a conventional relative risk measure such as a risk ratio, odds ratio, or hazard ratio. The RaSR and its confidence interval (CI) were analyzed on the log scale using the method proposed by Altman and Bland [21]. Because we evaluated multiple outcomes in parallel, we controlled the false discovery rate (FDR) using the Benjamini–Hochberg (BH) method, which involves ordering p-values and determining significance by comparing each p-value with a rank-specific threshold [22]. The FDR correction was applied separately within each group of hypotheses corresponding to the analysis models, including disease-category outcomes (autoimmune rheumatic disease, inflammatory bowel disease, and autoimmune endocrine disease) and individual autoimmune diseases. We presented BH-adjusted p-values for all outcomes, and we considered associations to be statistically significant if the BH-adjusted p-value was below 0.05. All analyses were performed using the SAS Enterprise Guide (version 7.1; SAS Institute Inc., Cary, NC, USA). The SSA was implemented using custom programming code rather than standard macros or packages.

### Ethical considerations

This study was approved by the Institutional Review Board (IRB) of Chung-Ang University Hospital (IRB approval number: 2209-016-19436). The requirement for informed consent was waived as the data used anonymized data and had a retrospective design. The study was conducted in accordance with the principles of the Declaration of Helsinki.

## Results

### Baseline demographic characteristics of the study population

**Fig 1:**
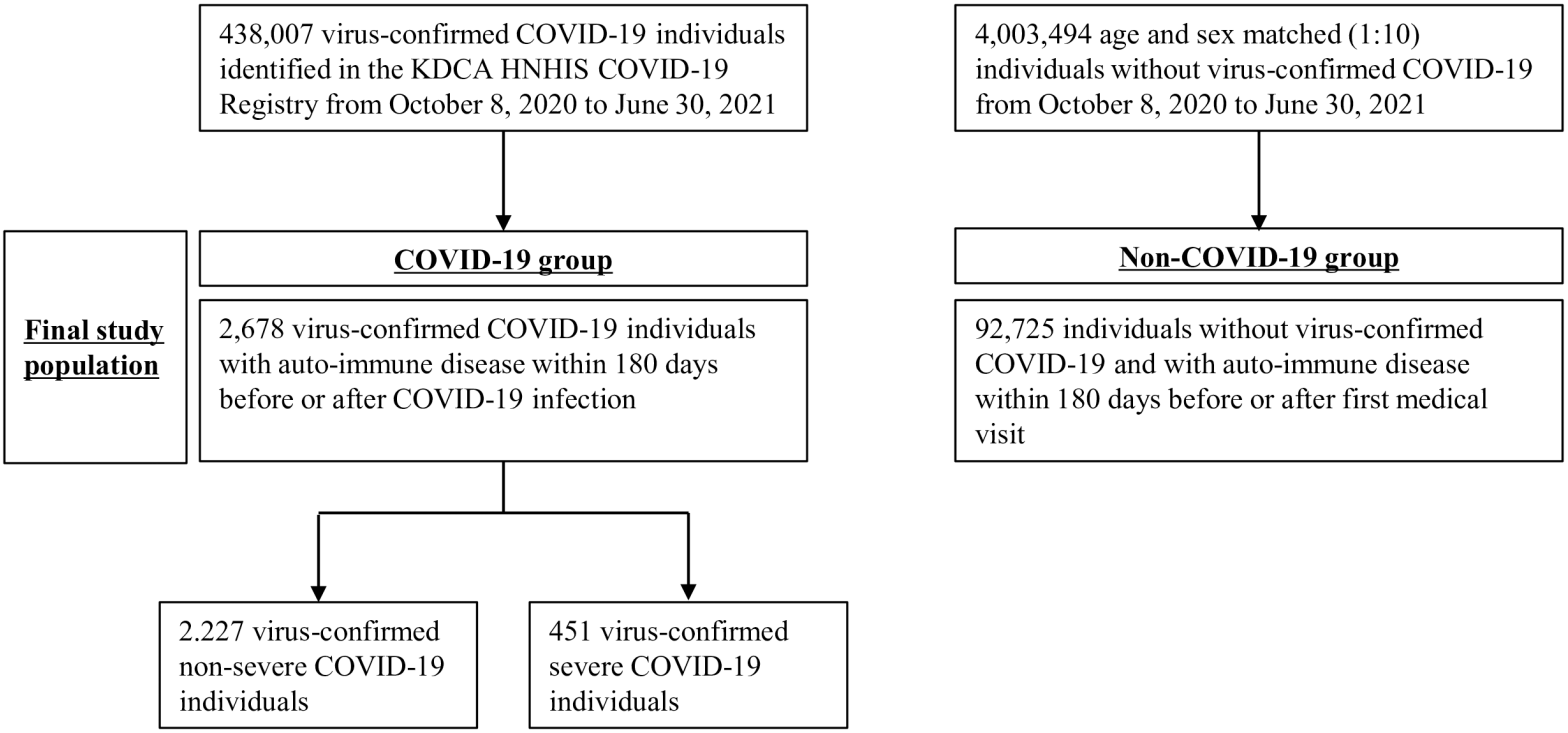
Flowchart of the selection of study participants.

In the KCDA-NHIS COVID-19 registry, a total of 438,007 individuals with laboratory-confirmed COVID-19 were identified between 01/10/2020 and 30/06/2021. For SSA, we included patients who had a diagnosis of autoimmune disease within 180 days before and after the index date: 2,678 patients in the COVID-19 group and 92,725 individuals in the non-COVID-19 group (Fig 1). The baseline characteristics of the study population are described in Table 1. The mean ages of patients in the COVID-19 and non-COVID-19 groups were 55.4 and 54.8 years, respectively. Approximately two-thirds of patients were female (62.3% in the COVID-19 group and 63.6% in the non-COVID-19 group). The majority of patients in both groups has a Charlson comorbidity index score of 0–2. Detailed information on underlying comorbidities is presented in S2 Table. Mild liver disease (COVID-19, 11.9% versus non-COVID-19, 11.2%) and DM without complications (11.1% vs. 9.7%) were the most common underlying conditions in both groups.

**Table 1:** Baseline characteristics of the study population in sequence symmetry analysis.

	COVID-19 (n = 2,678)	Non-COVID-19 (n = 92,725)
**Age, mean ± SD**	55.4 ± 15.7	54.8 ± 15.9
≤19	17 (0.6)	57 (0.9)
20–39	448 (16.7)	801 (18.9)
40–49	414 (15.5)	17 477 (14.7)
50–59	635 (23.7)	13,669 (21.8)
60–69	690 (25.8)	20,168 (26.3)
70–79	327 (12.2)	11,803 (12.7)
80≥	147 (5.5)	4,416 (4.8)
**Sex**		
Male	1,010 (37.7)	33,721 (36.4)
Female	1,668 (62.3)	59,004 (63.6)
**Charlson comorbidity index, mean ± SD**	0.6 ± 1.3	0.5 ± 1.2
0–2	2,461 (91.9)	86,467 (93.3)
3–5	188 (7.0)	5,412 (5.8)
≥6	29 (1.1)	846 (0.9)

COVID-19, coronavirus disease 2019; SD, standard deviation.

Data are presented as No. (%) unless otherwise stated.

### Association between COVID-19 and new-onset autoimmune diseases

**Fig 2:**
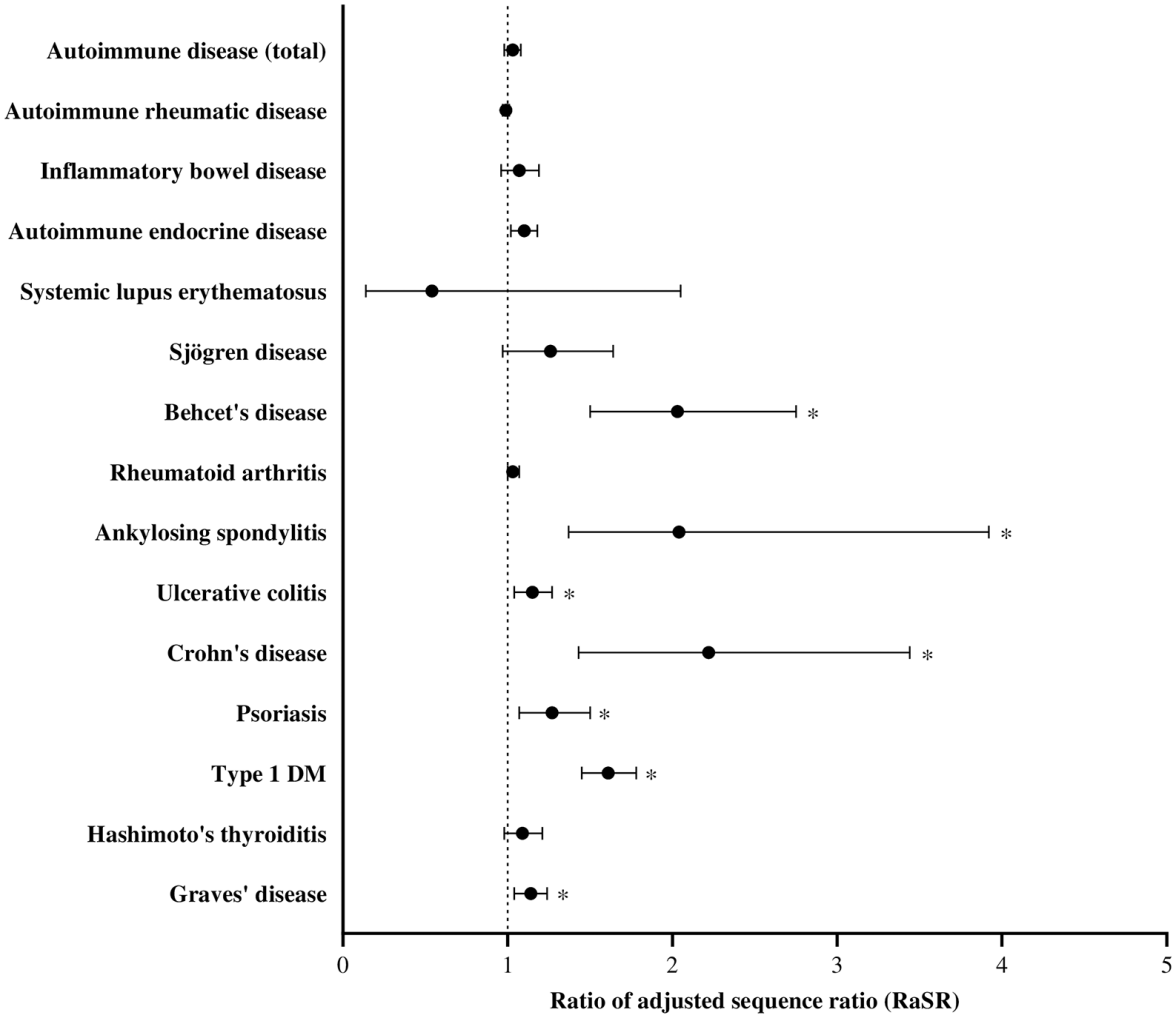
Ratio of adjusted sequence ratio for new-onset autoimmune diseases before and after the index date between COVID-19 and non-COVID-19 group. *An asterisk indicates statistical significance after Benjamini-Hochberg adjustment (adjusted p < 0.05).

We performed SSA to compare the incidence of autoimmune disease before and after the index date between the COVID-19 and non-COVID-19 groups (Fig 2 and S3 Table). The incidence of BD, AS, UC, CD, psoriasis, type 1 DM, and Graves’ disease was significantly higher in the COVID-19 group than that in the non-COVID-19 group; the corresponding RaSRs and BH-adjusted p-values are as follows: BD: 2.03 (p < 0.01); AS: 2.04 (p = 0.02); UC: 1.15 (p = 0.02); CD: 2.22 (p < 0.01); psoriasis: 1.27 (p = 0.01); type I DM, 1.61 (p < 0.01); and Graves’ disease: 1.14 (p = 0.01). To validate these findings, we performed a sensitivity analysis based on variations in the time interval (*d*) and study duration before and after the index date (S4 Table). The RaSR results were largely consistent with the primary findings. The incidence of CD (1.93 (p < 0.01) with time interval 14 days and duration before/after index date 180 days; 6.54 (p < 0.01) with time interval 28 days and duration before/after index date 120 days; 3.94 (p < 0.01) with time interval 14 days and duration before/after index date 120 days), BD (1.32 (p < 0.01); 2.69 (p < 0.01); 1.71 (p < 0.01)), type 1 DM (1.49 (p < 0.01); 2.27 (p < 0.01); 2.02 (p < 0.01)) were significantly higher in the COVID-19 group than that in the non-COVID-19 group in all sensitivity analyses. In addition, the incidence rate of autoimmune disease within 180 days in the COVID-19 and the non-COVID-19 groups after the index date is shown in the S5 Table.

### Subgroup analysis

**Fig 3:**
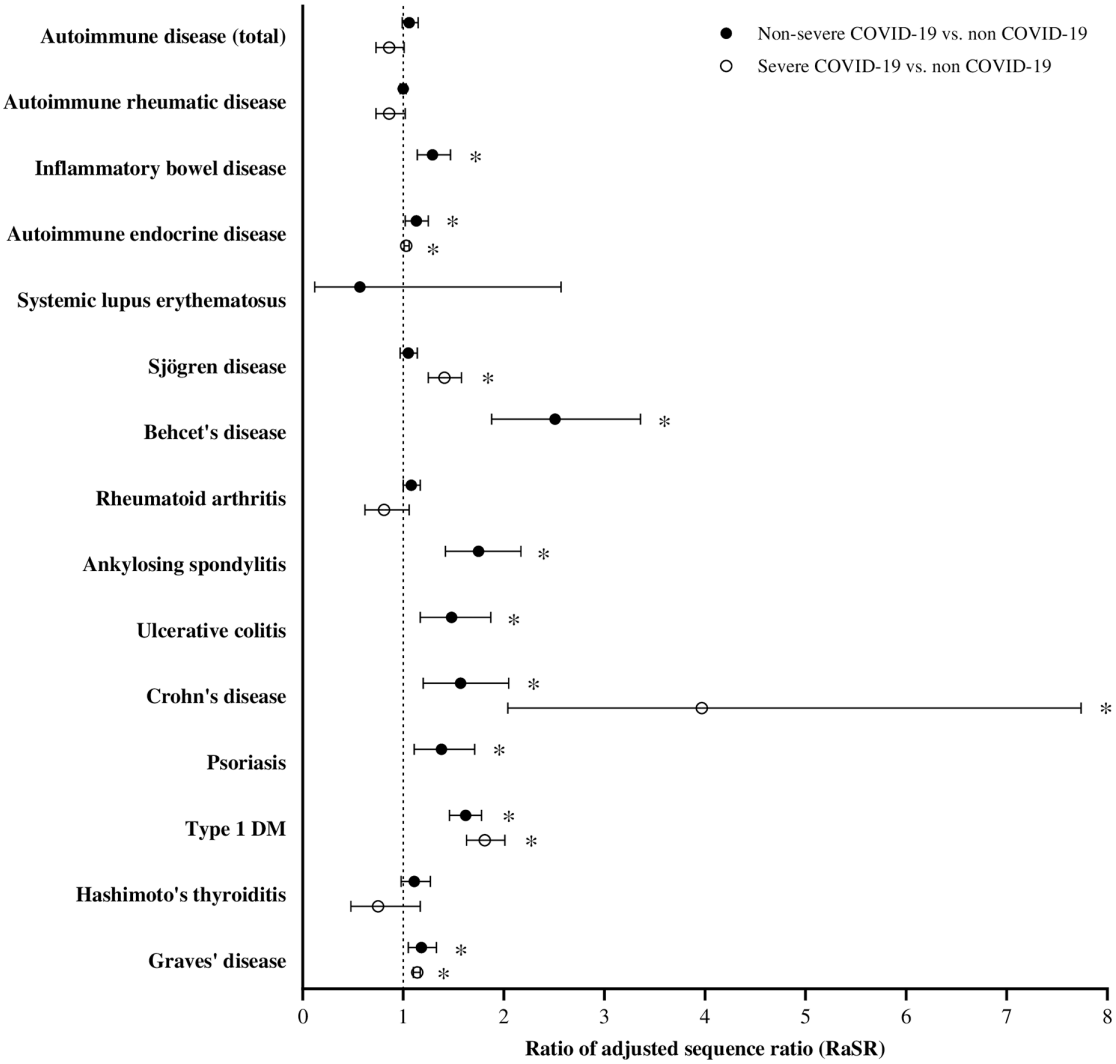
Ratio of adjusted sequence ratio for new-onset autoimmune diseases before and after the index date between non-severe COVID-19, severe COVID-19 and non-COVID-19 groups. *An asterisk indicates statistical significance after Benjamini-Hochberg adjustment (adjusted p < 0.05).

We performed a subgroup analysis to compare the incidence of autoimmune diseases before and after the index date between the non-severe COVID-19 and non-COVID-19 groups (Fig 3 and S6 Table). The results in the non-severe COVID-19 group were largely consistent with those of the overall population. Notably, the incidence of inflammatory bowel diseases was significantly higher in the non-severe COVID-19 group than that in the non-COVID-19 group (RaSR, 1.29, p < 0.01). A subgroup analysis was also performed for individuals in the severe COVID-19 group. However, due to the limited number of new-onset autoimmune disease cases in this subgroup, RaSR estimates could not be calculated for most diseases. Statistically significant results were observed only for SjD, CD, type 1 DM, and Grave’s disease (Fig 3 and S6 Table).

## Discussion

In this study, we conducted SSA to evaluate the differences in the incidence of autoimmune diseases before and after the index date among 2,678 patients with COVID-19 and 92,725 patients without COVID-19. The analysis revealed that the incidence of newly diagnosed BD, AS, UC, CD, psoriasis, type 1 DM, and Graves’ disease was significantly higher in the COVID-19 group than that in the non-COVID-19 group after the index date. Subgroup analysis comparing patients with non-severe COVID-19 with those without COVID-19 yielded results consistent with the main findings. Additionally, the incidence of inflammatory bowel diseases was significantly higher in the non-severe COVID-19 group than that in the non-COVID-19 group.

The development of autoimmune diseases is attributed to abnormal immune responses, wherein the immune system fails to differentiate self-antigens from non-self-antigens. The etiology of autoimmune diseases is multifactorial, including genetic susceptibility, age, and environmental factors. Viruses have been recognized as a significant environmental trigger of autoimmune diseases [1,2,9]. Several mechanisms have been proposed to explain how viral infections can disrupt self-tolerance [23,24]. First, as viruses express antigens that are structurally similar to self-antigens, a phenomenon called “molecular mimicry” triggers cross-reactive immune responses against self-antigens [25–28]. Second, “bystander activation” refers to non-specific and excessive antiviral immune responses that create localized proinflammatory environments. This phenomenon promotes the release of self-antigens from the damaged tissue, and, when presented on antigen presenting cells, these self-antigens can activate autoreactive T cells [29]. Third, “epitope spreading” involves the release of self-antigens and activation of autoreactive cells produced as a result of the above two mechanisms. These cells subsequently expand to target additional self-antigens and activate more autoreactive T cells, amplifying the autoimmune response [24]. Additionally, certain viruses may contribute to autoimmunity by immortalizing autoreactive cells [30]. Similar mechanisms have been proposed to explain SARS-CoV-2–associated autoimmunity. Various autoantibodies, including antinuclear antibodies, lupus anticoagulant cold agglutinins, and anti-Ro/SSA antibodies, were detected in patients with COVID-19 [10,31]. Additionally, SARS-CoV-2 can affect multiple organs beyond the lung owing to the widespread expression of its entry receptors, angiotensin converting enzyme-2 (ACE-2), and transmembrane serine protease 2 (TMPRSS2) [32,33]. Consequently, concerns have emerged regarding the potential of SARS-CoV-2 to induce or exacerbate autoimmune diseases among infected individuals.

The details of the current study along with prior research [3–8] on the relationship between COVID-19 and autoimmune diseases are presented in S7 Table. Previous studies have compared the incidence of autoimmune diseases between patients with COVID-19 and matched controls. Although the results varied, the overall trend indicated an increasing incidence of autoimmune diseases following COVID-19. In this study, the incidence of type 1 DM and Graves’ disease were significantly higher in the COVID-19 group than that in the non-COVID-19 group after the index date, aligning with that reported in previous studies [9]. Because ACE-2 and TMPRSS2 are widely expressed in many endocrine glands such as the pancreas and thyroid gland, the endocrine system is vulnerable to both destruction and alteration in function following COVID-19 [34].

Similarly, we observed an increased incidence of UC and CD after the index date among patients with COVID-19, a finding consistent with that reported in the literature [4,6,7]. Given the high expression of ACE-2 in the intestine, it is plausible that SARS-CoV-2 infection may contribute to the development or exacerbation of gastrointestinal diseases such as inflammatory bowel diseases. Furthermore, CD is primarily associated with Th1 and Th17 cytokine profiles, whereas UC is linked to Th2 cytokines [35]. Dysregulated Th1 immune responses may occur in patients with COVID-19, potentially leading to cytokine storms that subsequently trigger excessive Th2 immune activation. Therefore, these phenomena could further support the association between COVID-19 and inflammatory bowel disease.

In this study, patients with COVID-19 demonstrated a significantly higher risk of psoriasis than those without. Given that psoriasis is a T cell-mediated chronic inflammatory skin disease [36], this phenomenon may similarly be related to SARS-CoV-2–induced dysregulated T-cell immune responses. The incidence of AS was also higher among patients with COVID-19, which is consistent with previous reports indicating that COVID-19 infection can be associated with the development of AS [37–40]. Several mechanisms have been proposed to explain this association. Antigen-specific T-cell responses during COVID-19 infection may induce AS-associated autoreactive T cells, leading to a plausible trade-off between the benefits of immunological host defense and the risk of autoimmunity [37]. Additionally, molecular mimicry involving SARS-CoV-2 and genetic susceptibility—particularly HLA-B27 positivity—may contribute to the heightened susceptibility to COVID-19–related spondyloarthritis [38,40]. Consistent with previous reports suggesting a potential association between COVID-19 infection and the development of BD [3,4,41], the incidence of BD also increased among patients with COVID-19 in this study. Gene–transcription factor–miRNA networks, gene–disease networks, and gene–drug networks have been proposed as possible mechanisms underlying the interaction between BD and COVID-19 [42]. Nevertheless, because the mechanistic explanations for BD remain incomplete, it is possible that the observed association may reflect ascertainment bias.

One of the main strengths of this study is the methodological approach with SSA, which is an extension of the case-crossover design. In this study, all patients served as their own controls, experiencing both the exposure (COVID-19 diagnosis or hospital visit) and the outcome (new-onset autoimmune disease), thus minimizing time-invariant confounding factors such as genetic susceptibility and underlying medical conditions. In addition, we applied neSR adjustment for temporal trends in autoimmune disease incidence. Furthermore, the comparative analysis of the two estimated ratios between patients with and without COVID-19 enabled an intuitive and clinically meaningful interpretation about the relationship between COVID-19 and subsequent autoimmune disease incidence. Additionally, we included several autoimmune diseases in the analysis to enhance the generalizability and robustness of our findings.

Despite these strengths, this study has some limitations. First, as the study period (01/10/2020 to 30/06/2021) corresponds to the early phase of the COVID-19 pandemic—before widespread vaccine distribution and the emergence of Delta and Omicron variants. During this period, South Korea implemented stringent national infection control policies. As a result, we could not adequately evaluate the impact of the vaccination status, viral variants, and COVID-19 severity on the development of autoimmune diseases. Previous reports have shown that SARS-CoV-2 vaccination reduce the risk of new-onset autoimmune diseases [5,8], severe COVID-19 increases autoimmune disease risk [4,8], and infection with Omicron variant may be associated with a lower risk [7]. Further studies evaluating these factors is therefore warranted. Second, autoimmune diseases were identified using claims-based definitions without clinical or laboratory confirmation, which may have led to either overestimation or underestimation of autoimmune diseases. In addition, although undetected infections could have introduced bias, the rigorous and widely applied national testing policy during the early pandemic likely minimized such misclassification in South Korea. Third, the follow-up period was limited as 180 days. Because some autoimmune diseases may develop over several months to years, longer observation periods are necessary to capture delayed-onset autoimmune diseases. However, extending the follow-up period could also introduce additional triggers, such as other viral infections and environmental exposures, that are unrelated to the index COVID-19 episode. These competing factors may confound the causal association between COVID-19 and subsequent autoimmune diseases. Thus, future studies with longer follow-up periods should incorporate analytical strategies to address these potential confounders. Fourth, subgroup analyses considering other confounding factors such as genetic, hormonal, or other immunologic factors could not be performed because of data unavailability. Another limitation is low statistical power for rare autoimmune diseases. Although the overall cohort was large, some disease had few incident cases, which limits the ability of the SSA analysis to detect asymmetry in the event sequence. Therefore, non-significant findings for rare autoimmune diseases should be interpreted with caution, as they may reflect limited statistical power due to small event counts rather than the absence of an association.

Finally, as this study was performed within a single national healthcare system during the early pandemic period, the findings may not be fully generalizable to all COVID-19 patients. Nevertheless, this study provides supporting evidence for an increased incidence of autoimmune diseases following COVID-19 through an alternative statistical approach called SSA. SSA offers a methodological advantage by inherently adjusting for time-invariant confounding factors, which may be overlooked in traditional matched cohort designs. However, there are still concerns about time-varying confounders, such as changes in healthcare utilization, diagnostic intensity could not be fully accounted for and may still have influenced the results. Therefore, further studies incorporating these additional factors in conjunction with SSA are warranted to validate the associations observed in this study.

## Conclusion

In conclusion, among individuals who received a diagnosis of autoimmune disease within 180 days before or after the index date, we observed a higher likelihood of autoimmune disease being diagnosed after rather than before COVID-19, notably BD, AS, UC, CD, psoriasis, type 1 DM, and Graves’ disease. These findings reinforce growing evidence that COVID-19 could induce autoimmunity and increase the risk of developing autoimmune diseases. Therefore, clinicians should remain vigilant for potential autoimmune complications in patients recovering with a history of COVID-19 when clinically indicated. Future studies examining the impacts of different SARS-CoV-2 variants, disease severity, and vaccination status on the risk of autoimmune diseases are warranted.

## Supporting information

S1 Table International Classification of Diseases (ICD)-10-based codes for autoimmune diseases. (DOCX)

S2 Table Underlying diseases of the study population in sequence symmetry analysis. (DOCX)

S3 Table Sequence symmetry ratio for the diagnosis of autoimmune disease between the COVID-19 and non-COVID-19 groups (time interval 28 days, duration before/after index date 180 days). (DOCX)

S4 Table Sensitivity analysis according to the time intervaland study duration before and after the index date. (A) Sequence symmetry ratio for the diagnosis of autoimmune disease between the COVID-19 and non-COVID-19 groups (time interval14 days, duration before/after the index date 180 days). (B) Sequence symmetry ratio for the diagnosis of autoimmune disease between the COVID-19 and non-COVID-19 groups (time interval28 days, duration before/after index date 120 days). (C) Sequence symmetry ratio for the diagnosis of autoimmune disease between the COVID-19 and non-COVID-19 groups (time interval14 days, duration before/after index date 120 days)
(DOCX)

S5 Table Autoimmune disease events within 180 Days in COVID19/non-COVID-19 groups after the index date. (DOCX)

S6 Table (A) Sequence symmetry ratio for the diagnosis of autoimmune disease between the non-severe COVID-19 and non-COVID-19 groups. (B) Sequence symmetry ratio for the diagnosis of autoimmune disease between the severe COVID-19 and non-COVID-19 groups
(DOCX)

S7 Table Summary of previous studies on the association between COVID-19 and incident autoimmune diseases. (DOCX)
