# Supplementary material for: Association between coronavirus disease 2019 and new-onset autoimmune diseases during the early phase of the pandemic
Source: PLoS One. 2026 May 5;21(5):e0347872. doi: 10.1371/journal.pone.0347872 (PMC13143056; doi:10.1371/journal.pone.0347872)
Supplement: S1 Table — (DOCX) [file pone.0347872.s001.docx]

**S1 Table.** **International Classification of Diseases (ICD)-10-based codes for autoimmune diseases**

| **Autoimmune diseases** | **ICD-10 code** |
| --- | --- |
| Systemic lupus erythematosus | M32 |
| Systemic sclerosis | M34 |
| Idiopathic inflammatory myopathy | M33 |
| Sjögren disease | M350 |
| Mixed connective tissue disease | M351 |
| Behcet’s disease | M352 |
| Polymyalgia rheumatica | M353 |
| Rheumatoid arthritis | M05, M06 |
| Ankylosing spondylitis | M450 |
| Adult-onset Still’s disease | V06, V298 |
| Ulcerative colitis | K51 |
| Crohn’s disease | K50 |
| Autoimmune hepatitis | L754 |
| Granulomatosis with polyangiitis | M313 |
| Microscopic polyangiitis | M317 |
| Eosinophilic granulomatosis with polyangiitis | M301 |
| Polyarteritis nodosa | M300 |
| Takayasu arteritis | M314 |
| Multiple sclerosis | G35 |
| Psoriasis | L40 |
| Type 1 diabetes mellitus | E10 |
| Hashimoto | E06 |
| Graves’ disease | E05 |
