# Supplementary material for: Association between coronavirus disease 2019 and new-onset autoimmune diseases during the early phase of the pandemic
Source: PLoS One. 2026 May 5;21(5):e0347872. doi: 10.1371/journal.pone.0347872 (PMC13143056; doi:10.1371/journal.pone.0347872)
Supplement: S2 Table — (DOCX) [file pone.0347872.s002.docx]

**S2 Table. Underlying diseases of the study population in sequence symmetry analysis**

|  | **COVID-19**  **(n = 2,678)** | | **Non-COVID-19**  **(n = 92,725)** |
| --- | --- | --- | --- |
| **Underlying diseases, n (%)** |  |  |  |
| Mild liver disease | 318 (11.9) | | 10,399 (11.2) |
| Diabetes mellitus without complications | 297 (11.1) | | 8,986 (9.7) |
| Diabetes mellitus with complications | 84 (3.1) | | 2,657 (2.9) |
| Malignancy | 75 (2.8) | | 2,418 (2.6) |
| Peptic ulcer disease | 71 (2.7) | | 2,166 (2.3) |
| Congestive heart failure | 68 (2.5) | | 1,102 (1.2) |
| Peripheral vascular disease | 62 (2.3) | | 1,667 (1.8) |
| Chronic pulmonary disease | 59 (2.2) | | 1,665 (1.8) |
| Renal disease | 47 (1.8) | | 1,150 (1.2) |
| Dementia | 24 (0.9) | | 511 (0.6) |
| Cerebrovascular disease | 19 (0.7) | | 476 (0.5) |
| Myocardial infarction | 11 (0.4) | | 260 (0.3) |
| Moderate or severe liver disease | 5 (0.2) | | 82 (0.1) |
| Metastatic solid tumor | 2 (0.1) | | 214 (0.2) |
| Hemiplegia or paraplegia | 1 (0.0) | | 99 (0.1) |
| HIV/AIDS | 1 (0.0) | | 31 (0.0) |

COVID-19, coronavirus disease 2019; HIV/AIDS, human immunodeficiency virus/acquired immunodeficiency syndrome

Data are presented as No. (%) unless otherwise stated.
