# Supplementary material for: Association between coronavirus disease 2019 and new-onset autoimmune diseases during the early phase of the pandemic
Source: PLoS One. 2026 May 5;21(5):e0347872. doi: 10.1371/journal.pone.0347872 (PMC13143056; doi:10.1371/journal.pone.0347872)
Supplement: S3 Table — (DOCX) [file pone.0347872.s003.docx]

**S3 Table. Sequence symmetry ratio for the diagnosis of autoimmune disease between the COVID-19 and non-COVID-19 groups (time interval28 days, duration before/after index date 180 days)**

| **Diagnosis** | **COVID-19** | | | | | **Non-COVID-19** | | | | | **Difference**  **between log(aSR)** | |
| --- | --- | --- | --- | --- | --- | --- | --- | --- | --- | --- | --- | --- |
|  | **Before** | **After** | **neSR** | **aSR**  **(95% CI)** | **BH-adjusted *p*-value** | **Before** | **After** | **neSR** | **aSR**  **(95% CI)** | **BH-adjusted *p*-value** | **RaSR**  **(95% CI)** | **BH-adjusted *p*-value** |
| **Autoimmune disease** | 1,310 | 1,368 | 1.00 | 1.05 (0.97–1.12) | 0.23 | 43,821 | 48,904 | 1.10 | 1.02 (1.00–1.03) | 0.01 | 1.03 (0.98–1.08) | 0.20 |
| **Autoimmune rheumatic disease** | 638 | 639 | 0.99 | 1.01 (0.90–1.12) | 1.00 | 20,576 | 23,789 | 1.13 | 1.03 (1.01–1.05) | 0.02 | 0.99 (0.97–1.01) | 1.00 |
| **Inflammatory bowel disease^a^** | 36 | 42 | 1.15 | 1.02 (0.57–1.46) | 0.95 | 1,093 | 1,382 | 1.33 | 0.95 (0.87–1.03) | 0.60 | 1.07 (0.96–1.19) | 0.96 |
| **Autoimmune endocrine disease^b^** | 548 | 599 | 1.01 | 1.08 (0.97–1.20) | 0.42 | 17,970 | 19,695 | 1.11 | 0.99 (0.97–1.01) | 0.40 | 1.10 (1.02–1.18) | 0.21 |
| **Systemic lupus erythematosus** | 71 | 31 | 0.90 | 0.49 (0.06–0.91) | 0.57 | 1,166 | 1,443 | 1.38 | 0.90 (0.82–0.97) | 0.01 | 0.54 (0.14–2.05) | 0.43 |
| **Systemic sclerosis** | 5 | 1 | 0.43 | 0.47 (<0.0001–2.61) | 0.98 | 95 | 115 | 2.03 | 0.60 (0.33–0.87) | 0.01 | 0.78 (0.01–49.8) | 0.84 |
| **Idiopathic inflammatory myopathy** | 5 | 3 | 0.54 | 1.11 (<0.0001–2.55) | 1.00 | 129 | 176 | 1.99 | 0.69 (0.46–0.91) | 0.01 | 1.62 (0.03–103.3) | 0.82 |
| **Sjögren disease** | 27 | 35 | 0.94 | 1.38 (0.87–1.88) | 0.36 | 841 | 1,407 | 1.54 | 1.09 (1.00–1.17) | 0.01 | 1.26 (0.97–1.64) | 0.11 |
| **Mixed connective tissue disease** | 2 | 1 | . | . |  | 71 | 63 | 3.30 | 0.27 (<0.0001–0.61) | 0.13 |  |  |
| **Behcet’s disease** | 10 | 9 | 0.73 | 1.24 (0.34–2.14) | 1.00 | 303 | 366 | 1.98 | 0.61 (0.46–0.76) | <.01 | 2.03 (1.50–2.75) | <.01 |
| **Polymyalgia rheumatica** | 4 | 6 | 1.50 | 1.00 (<0.0001–2.27) | 1.00 | 108 | 152 | 2.97 | 0.47 (0.23–0.72) | 0.01 | 2.11 (0.03–146.0) | 0.79 |
| **Rheumatoid arthritis** | 542 | 559 | 0.99 | 1.04 (0.92–1.16) | 0.84 | 18,430 | 21,073 | 1.13 | 1.01 (0.99–1.03) | 0.09 | 1.03 (1.00–1.07) | 0.13 |
| **Ankylosing spondylitis** | 13 | 20 | 0.96 | 1.61 (0.91–2.30) | 0.21 | 483 | 608 | 1.60 | 0.79 (0.67–0.91) | <.01 | 2.04 (1.37–3.92) | 0.02 |
| **Adult-onset Still’s disease** | 1 | 2 | . | . |  | 25 | 15 | 9.11 | 0.07 (<0.0001–0.71) | 0.06 |  |  |
| **Ulcerative colitis** | 26 | 29 | 1.04 | 1.07 (0.54–1.60) | 0.93 | 747 | 969 | 1.39 | 0.94 (0.84–1.03) | 0.06 | 1.15 (1.04–1.27) | 0.02 |
| **Crohn’s disease** | 11 | 17 | 0.92 | 1.67 (0.91–2.43) | 0.55 | 369 | 457 | 1.64 | 0.75 (0.62-0.89) | <.01 | 2.22 (1.43–3.44) | <.01 |
| **Autoimmune hepatitis** | 1 | 1 | . | . |  | 52 | 78 | 2.92 | 0.51 (0.16–0.86) | 0.03 |  |  |
| **Granulomatosis with polyangiitis** | . | . | . | . |  | 14 | 24 | 4.06 | 0.42 (<0.0001–1.08) | 0.16 |  |  |
| **Microscopic polyangiitis** | 0 | 2 | . | . |  | 10 | 23 | 0.92 | 2.50 (1.75–3.24) | <.01 |  |  |
| **Eosinophilic granulomatosis with polyangiitis** | . | . | . | . |  | 8 | 12 | 0.25 | 6.00 (5.11–6.89) | <.01 |  |  |
| **Polyarteritis nodosa** | . | . | . | . |  | 10 | 13 | 5.07 | 0.26 (<0.0001–1.08) | 0.13 |  |  |
| **Takayasu’s arteritis** | 0 | 1 | . | . |  | 15 | 16 | 0.63 | 1.70 (0.99–2.40) | 0.01 |  |  |
| **Multiple sclerosis** | 0 | 4 | . | . |  | 108 | 135 | 2.48 | 0.50 (0.25–0.76) | 0.01 |  |  |
| **Psoriasis** | 113 | 135 | 1.00 | 1.20 (0.95–1.45) | 0.42 | 4,796 | 5,342 | 1.18 | 0.95 (0.91–0.99) | 0.01 | 1.27 (1.07–1.50) | 0.01 |
| **Type 1 diabetes mellitus** | 68 | 70 | 0.96 | 1.08 (0.74–1.41) | 0.91 | 1,823 | 1,756 | 1.44 | 0.67 (0.60–0.73) | <.01 | 1.61 (1.45–1.78) | <.01 |
| **Hashimoto’s disease** | 267 | 292 | 0.97 | 1.12 (0.96–1.29) | 0.36 | 8,656 | 9,919 | 1.11 | 1.03 (1.00–1.06) | 0.02 | 1.09 (0.98–1.21) | 0.15 |
| **Graves’ disease** | 235 | 263 | 1.02 | 1.10 (0.92–1.27) | 0.60 | 8,480 | 9,252 | 1.13 | 0.97 (0.94–1.00) | 0.01 | 1.14 (1.04–1.24) | 0.01 |

COVID-19, coronavirus 2019; neSR, null-effect sequence ratio; aSR, adjusted sequence ratio; CI, confidence interval; BH-adjusted p-value, Benjamini-Hochberg-adjusted p-value
