# Supplementary material for: Association between coronavirus disease 2019 and new-onset autoimmune diseases during the early phase of the pandemic
Source: PLoS One. 2026 May 5;21(5):e0347872. doi: 10.1371/journal.pone.0347872 (PMC13143056; doi:10.1371/journal.pone.0347872)
Supplement: S4 Table — (A) Sequence symmetry ratio for the diagnosis of autoimmune disease between the COVID-19 and non-COVID-19 groups (time interval14 days, duration before/after the index date 180 days). (B) Sequence symmetry ratio for the diagnosis of autoimmune disease between the COVID-19 and non-COVID-19 groups (time interval28 days, duration before/after index date 120 days). (C) Sequence symmetry ratio for the diagnosis of autoimmune disease between the COVID-19 and non-COVID-19 groups (time interval14 days, duration before/after index date 120 days) (DOCX) [file pone.0347872.s004.docx]

**S4 Table. Sensitivity analysis according to the exposure window and study duration before and after the index date**

**(A) Sequence symmetry ratio for the diagnosis of autoimmune disease between the COVID-19 and non-COVID-19 groups (time interval 14 days, duration before/after the index date 180 days)**

| **Diagnosis** | **COVID-19** | | | | | **Non-COVID-19** | | | | | **Difference**  **between log(aSR)** | |
| --- | --- | --- | --- | --- | --- | --- | --- | --- | --- | --- | --- | --- |
|  | **Before** | **After** | **neSR** | **aSR**  **(95% CI)** | **BH-adjusted *p*-value** | **Before** | **After** | **neSR** | **aSR**  **(95% CI)** | **BH-adjusted *p*-value** | **RaSR**  **(95% CI)** | **BH-adjusted *p*-value** |
| **Autoimmune disease** | 1,310 | 1,368 | 1.00 | 1.05 (0.97–1.12) | 0.21 | 43,821 | 48,904 | 1.08 | 1.03 (1.02–1.05) | <.01 | 1.01 (0.97–1.06) | 0.56 |
| **Autoimmune rheumatic disease** | 638 | 639 | 0.99 | 1.01 (0.90–1.12) | 1.00 | 20,576 | 23,789 | 1.09 | 1.06 (1.04–1.08) | <.01 | 0.96 (0.94–0.98) | <.01 |
| **Inflammatory bowel disease^a^** | 36 | 42 | 1.11 | 1.05 (0.61–1.50) | 0.83 | 1,093 | 1,382 | 1.22 | 1.04 (0.96–1.12) | 0.52 | 1.01 (0.96–1.08) | 0.65 |
| **Autoimmune endocrine disease^b^** | 25 | 40 | 0.95 | 1.69 (1.19–2.19) | <.01 | 17,970 | 19,695 | 1.09 | 1.01 (0.99–1.03) | 0.38 | 1.68 (1.24–2.27) | <.01 |
| **Systemic lupus erythematosus** | 71 | 31 | 0.91 | 0.48 (0.06–0.90) | 0.55 | 1,166 | 1,443 | 1.26 | 0.98 (0.91–1.06) | 0.73 | 0.49 (0.12–1.93) | 0.43 |
| **Systemic sclerosis** | 5 | 1 | 0.67 | 0.30 (<0.0001–2.45) | 0.83 | 95 | 115 | 1.43 | 0.84 (0.57–1.12) | 0.46 | 0.36 (0.01–22.8) | 1.00 |
| **Idiopathic inflammatory myopathy** | 5 | 3 | 0.43 | 1.40 (<0.0001–2.83) | 0.97 | 129 | 176 | 1.67 | 0.82 (0.59–1.04) | 0.34 | 1.72 (0.03–102.8) | 0.97 |
| **Sjögren disease** | 27 | 35 | 0.94 | 1.38 (0.88–1.88) | 0.31 | 841 | 1,407 | 1.34 | 1.25 (1.16–1.33) | <.01 | 1.11 (0.85–1.44) | 0.11 |
| **Mixed connective tissue disease** | 2 | 1 |  |  |  | 71 | 63 | 2.33 | 0.38 (0.04–0.72) | 0.32 |  |  |
| **Behcet’s disease** | 10 | 9 | 0.91 | 0.99 (0.09–1.89) | 0.99 | 303 | 366 | 1.61 | 0.75 (0.60–0.90) | 0.02 | 1.32 (0.53–3.29) | <.01 |
| **Polymyalgia rheumatica** | 4 | 6 |  |  |  | 108 | 152 | 1.91 | 0.74 (0.49–0.98) | 0.24 |  |  |
| **Rheumatoid arthritis** | 542 | 559 | 0.98 | 1.05 (0.93–1.17) | 0.67 | 18,430 | 21,073 | 1.10 | 1.04 (1.02–1.06) | <.01 | 1.01 (0.96–1.05) | 0.08 |
| **Ankylosing spondylitis** | 13 | 20 | 0.94 | 1.64 (0.94–2.34) | 0.22 | 483 | 608 | 1.38 | 0.91 (0.79–1.03) | 0.32 | 1.80 (1.20–2.70) | 0.02 |
| **Adult-onset Still’s disease** | 1 | 2 |  |  |  | 25 | 15 | 2.55 | 0.24 (<0.0001–0.88) | 0.64 | 0.23 (0.00–18.1) |  |
| **Ulcerative colitis** | 26 | 29 | 1.07 | 1.04 (0.51–1.57) | 1.04 | 747 | 969 | 1.25 | 1.04 (0.94–1.13) | 0.61 | 1.01 (0.90–1.13) | 0.01 |
| **Crohn’s disease** | 11 | 17 | 0.90 | 1.71 (0.96–2.47) | 0.34 | 369 | 457 | 1.39 | 0.89 (0.75–1.03) | 0.35 | 1.93 (1.23–3.02) | <.01 |
| **Autoimmune hepatitis** | 1 | 1 |  |  |  | 52 | 78 | 2.14 | 0.70 (0.35–1.05) | 0.34 |  |  |
| **Granulomatosis with polyangiitis** |  |  |  |  |  | 14 | 24 | 3.61 | 0.48 (<0.0001–1.13) | 0.75 |  |  |
| **Microscopic polyangiitis** | 0 | 2 |  |  |  | 10 | 23 | 0.95 | 2.41 (1.67–3.15) | <.01 |  |  |
| **Eosinophilic granulomatosis with polyangiitis** |  |  |  |  |  | 8 | 12 | 1.00 | 1.50 (0.61–2.39) | 0.38 |  |  |
| **Polyarteritis nodosa** |  |  |  |  |  | 10 | 13 | 3.56 | 0.36 (<0.0001–1.19) | 0.70 |  |  |
| **Takayasu’s arteritis** | 0 | 1 |  |  |  | 15 | 16 | 0.72 | 1.48 (0.77–2.18) | 0.32 |  |  |
| **Multiple sclerosis** | 0 | 4 |  |  |  | 108 | 135 | 1.80 | 0.69 (0.44–0.95) | 0.20 |  |  |
| **Psoriasis** | 113 | 135 | 1.01 | 1.19 (0.94–1.44) | 0.29 | 4,796 | 5,342 | 1.13 | 0.99 (0.95–1.02) | 0.60 | 1.21 (1.04–1.40) | 0.01 |
| **Type 1 diabetes mellitus** | 68 | 70 | 0.93 | 1.10 (0.77–1.44) | 0.77 | 1,823 | 1,756 | 1.30 | 0.74 (0.67–0.80) | <.01 | 1.49 (1.35–1.66) | <.01 |
| **Hashimoto’s disease** | 267 | 292 | 0.96 | 1.14 (0.97–1.30) | 0.36 | 8,656 | 9,919 | 1.09 | 1.06 (1.03–1.08) | <.01 | 1.08 (0.95–1.22) | 0.15 |
| **Graves’ disease** | 235 | 263 | 1.01 | 1.10 (0.93–1.28) | 0.50 | 8,480 | 9,252 | 1.10 | 0.99 (0.96–1.02) | 0.71 | 1.11 (1.02–1.21) | 0.01 |

COVID-19, coronavirus 2019; neSR, null-effect sequence ratio; aSR, adjusted sequence ratio; CI, confidence interval; BH-adjusted p-value, Benjamini-Hochberg-adjusted p-value

**(B) Sequence symmetry ratio for the diagnosis of autoimmune disease between the COVID-19 and non-COVID-19 groups (time interval** **28 days, duration before/after index date 120 days)**

| **Diagnosis** | **COVID-19** | | | | | **Non-COVID-19** | | | | | **Difference**  **between log(aSR)** | |
| --- | --- | --- | --- | --- | --- | --- | --- | --- | --- | --- | --- | --- |
|  | **Before** | **After** | **neSR** | **aSR**  **(95% CI)** | **BH-adjusted *p*-value** | **Before** | **After** | **neSR** | **aSR**  **(95% CI)** | **BH-adjusted *p*-value** | **RaSR**  **(95% CI)** | **BH-adjusted *p*-value** |
| **Autoimmune disease** | 904 | 977 | 0.99 | 1.09 (1.00–1.18) | 0.05 | 27,402 | 32,594 | 1.09 | 1.09 (1.07–1.10) | <.01 | 1.00 (0.92–1.08) | 0.99 |
| **Autoimmune rheumatic disease** | 442 | 453 | 0.99 | 1.04 (0.90–1.17) | 0.59 | 12,979 | 15,784 | 1.11 | 1.10 (1.08–1.12) | <.01 | 0.94 (0.91–0.98) | <.01 |
| **Inflammatory bowel disease^a^** | 26 | 28 | 0.91 | 1.18 (0.64–1.71) | 0.77 | 720 | 962 | 1.27 | 1.05 (0.96–1.15) | 0.26 | 1.12 (1.04–1.20) | <.01 |
| **Autoimmune endocrine disease^b^** | 25 | 40 | 0.98 | 1.64 (1.14–2.14) | 0.03 | 11,153 | 13,052 | 1.10 | 1.07 (1.04–1.09) | <.01 | 1.53 (1.12–2.10) | 0.03 |
| **Systemic lupus erythematosus** | 56 | 24 | 0.90 | 0.48 (<0.0001–0.95) | 1.00 | 749 | 941 | 1.47 | 0.86 (0.76–0.95) | 0.02 | 0.56 (0.01–54.5) | 0.95 |
| **Systemic sclerosis** | 2 | 1 | 0.00 |  |  | 61 | 79 | 2.65 | 0.49 (0.15–0.82) | 0.19 |  |  |
| **Idiopathic inflammatory myopathy** | 3 | 2 | 1.00 | 0.67 (<0.0001–2.46) | 1.00 | 81 | 110 | 2.05 | 0.66 (0.38–0.95) | 0.18 | 1.01 (0.02–65.9) | 1.00 |
| **Sjögren disease** | 21 | 29 | 0.92 | 1.51 (0.95–2.07) | 0.17 | 522 | 904 | 1.60 | 1.08 (0.97–1.19) | 0.22 | 1.39 (0.99–1.97) | 0.12 |
| **Mixed connective tissue disease** | 2 | 1 |  |  |  | 53 | 50 | 3.08 | 0.31 (<0.0001–0.69) | 0.73 |  |  |
| **Behcet’s disease** | 6 | 7 | 0.69 | 1.69 (0.59–2.78) | 0.48 | 183 | 238 | 2.07 | 0.63 (0.43–0.82) | 0.02 | 2.69 (1.79–4.02) | <.01 |
| **Polymyalgia rheumatica** | 2 | 5 | 2.00 | 1.25 (<0.0001–2.89) | 1.00 | 75 | 103 | 3.42 | 0.40 (0.10–0.70) | 0.15 | 3.11 (0.05–203.99) | 0.97 |
| **Rheumatoid arthritis** | 367 | 390 | 1.01 | 1.05 (0.91–1.20) | 0.74 | 11,592 | 13,977 | 1.16 | 1.04 (1.02–1.07) | <.01 | 1.01 (0.96–1.06) | 0.83 |
| **Ankylosing spondylitis** | 10 | 11 | 1.13 | 0.98 (0.12–1.83) | 0.97 | 297 | 410 | 1.71 | 0.81 (0.66–0.96) | 0.08 | 1.21 (0.56–2.63) | 0.91 |
| **Adult-onset Still’s disease** | 1 | 2 |  |  |  | 15 | 12 | 32.25 | 0.02 (<0.0001–0.78) | 0.19 |  |  |
| **Ulcerative colitis** | 21 | 18 | 0.84 | 1.02 (0.39–1.65) | 1.00 | 493 | 682 | 1.47 | 0.94 (0.82–1.06) | 0.48 | 1.09 (0.86–1.37) | 0.89 |
| **Crohn’s disease** | 6 | 12 | 0.44 | 4.56 (3.58–5.54) | <.01 | 242 | 311 | 1.84 | 0.70 (0.53–0.86) | 0.02 | 6.54 (4.71–9.09) | <.01 |
| **Autoimmune hepatitis** | 0 | 1 |  |  |  | 34 | 54 | 3.19 | 0.50 (0.07–0.93) | 0.46 |  |  |
| **Granulomatosis with polyangiitis** |  |  |  |  |  | 11 | 17 | 4.09 | 0.38 (<0.0001–1.14) | 0.75 |  |  |
| **Microscopic polyangiitis** | 0 | 2 |  |  |  | 8 | 18 | 1.64 | 1.37 (0.54–2.20) | 0.53 |  |  |
| **Eosinophilic granulomatosis with polyangiitis** |  |  |  |  |  | 6 | 9 |  |  |  |  |  |
| **Polyarteritis nodosa** |  |  |  |  |  | 6 | 8 | 3.88 | 0.34 (<0.0001–1.40) | 0.77 |  |  |
| **Takayasu’s arteritis** | 0 | 1 |  |  |  | 13 | 10 | 1.29 | 0.60 (<0.0001–1.42) | 0.83 |  |  |
| **Multiple sclerosis** | 0 | 2 |  |  |  | 75 | 98 | 2.55 | 0.51 (0.21–0.81) | 0.14 |  |  |
| **Psoriasis** | 71 | 86 | 0.99 | 1.22 (0.91–1.54) | 0.42 | 2,859 | 3,449 | 1.22 | 0.99 (0.94–1.04) | 0.73 | 1.24 (1.04–1.47) | 0.04 |
| **Type 1 diabetes mellitus** | 41 | 56 | 0.94 | 1.45 (1.05–1.86) | 0.07 | 1,204 | 1,145 | 1.48 | 0.64 (0.56–0.72) | <.01 | 2.27 (1.66–3.10) | <.01 |
| **Hashimoto’s disease** | 189 | 210 | 1.03 | 1.08 (0.88–1.27) | 0.92 | 5,303 | 6,539 | 1.14 | 1.08 (1.05–1.12) | <.01 | 1.00 (0.93–1.07) | 1.00 |
| **Graves’ disease** | 171 | 183 | 1.00 | 1.07 (0.86–1.28) | 0.88 | 5,248 | 6,125 | 1.15 | 1.02 (0.98–1.05) | 0.50 | 1.06 (1.00–1.11) | 0.13 |

COVID-19, coronavirus 2019; neSR, null-effect sequence ratio; aSR, adjusted sequence ratio; CI, confidence interval; BH-adjusted p-value, Benjamini-Hochberg-adjusted p-value

**(C) Sequence symmetry ratio for the diagnosis of autoimmune disease between the COVID-19 and non-COVID-19 groups (time interval** **14 days, duration before/after index date 120 days)**

| **Diagnosis** | **COVID-19** | | | | | **Non-COVID-19** | | | | | **Difference**  **between log(aSR)** | |
| --- | --- | --- | --- | --- | --- | --- | --- | --- | --- | --- | --- | --- |
|  | **Before** | **After** | **neSR** | **aSR**  **(95% CI)** | **BH-adjusted *p*-value** | **Before** | **After** | **neSR** | **aSR**  **(95% CI)** | **BH-adjusted *p*-value** | **RaSR**  **(95% CI)** | **BH-adjusted *p*-value** |
| **Autoimmune disease** | 904 | 977 | 0.99 | 1.09 (1.00–1.18) | 0.05 | 27,402 | 32,594 | 1.09 | 1.09 (1.07–1.10) | <.01 | 1.00 (0.92–1.08) | 0.99 |
| **Autoimmune rheumatic disease** | 442 | 453 | 0.99 | 1.04 (0.90–1.17) | 0.59 | 12,979 | 15,784 | 1.11 | 1.10 (1.08–1.12) | <.01 | 0.94 (0.91–0.98) | <.01 |
| **Inflammatory bowel disease^a^** | 26 | 28 | 0.92 | 1.17 (0.63–1.70) | 0.81 | 720 | 962 | 1.27 | 1.05 (0.96–1.15) | 0.26 | 1.11 (1.04–1.18) | <.01 |
| **Autoimmune endocrine disease^b^** | 25 | 40 | 0.95 | 1.69 (1.19–2.19) | 0.01 | 11,153 | 13,052 | 1.10 | 1.07 (1.04–1.09) | <.01 | 1.58 (1.17–2.15) | 0.01 |
| **Systemic lupus erythematosus** | 56 | 24 | 0.90 | 0.48 (0.00–0.95) | 1.00 | 749 | 941 | 1.31 | 0.96 (0.86–1.05) | 0.49 | 0.50 (0.01–48.63) | 0.92 |
| **Systemic sclerosis** | 2 | 1 |  |  |  | 61 | 79 | 1.70 | 0.76 (0.43–1.09) | 0.47 |  |  |
| **Idiopathic inflammatory myopathy** | 3 | 2 | 0.75 | 0.89 (<0.0001–2.68) | 0.96 | 81 | 110 | 1.67 | 0.81 (0.53–1.10) | 0.46 | 1.09 (0.02–67.4) | 0.97 |
| **Sjögren disease** | 21 | 29 | 0.93 | 1.49 (0.93–2.05) | 0.20 | 522 | 904 | 1.38 | 1.26 (1.15–1.36) | <.01 | 1.19 (0.85–1.66) | 0.63 |
| **Mixed connective tissue disease** | 2 | 1 |  |  |  | 53 | 50 | 2.17 | 0.44 (0.05–0.82) | 0.50 |  |  |
| **Behcet’s disease** | 6 | 7 | 0.86 | 1.36 (0.27–2.45) | 0.88 | 183 | 238 | 1.63 | 0.80 (0.60–0.99) | 0.26 | 1.71 (1.24–2.36) | <.01 |
| **Polymyalgia rheumatica** | 2 | 5 |  |  |  | 75 | 103 | 2.20 | 0.62 (0.33–0.92) | 0.24 |  |  |
| **Rheumatoid arthritis** | 367 | 390 | 1.01 | 1.05 (0.91–1.20) | 0.77 | 11,592 | 13,977 | 1.11 | 1.08 (1.06–1.11) | <.01 | 0.97 (0.93–1.02) | 0.65 |
| **Ankylosing spondylitis** | 10 | 11 | 1.42 | 0.78 (<0.0001–1.63) | 1.00 | 297 | 410 | 1.42 | 0.97 (0.82–1.12) | 0.77 | 0.80 (0.01–62.4) | 1.00 |
| **Adult-onset Still’s disease** | 1 | 2 |  |  |  | 15 | 12 | 8.00 | 0.10 (-0.66–0.86) | 0.47 |  |  |
| **Ulcerative colitis** | 21 | 18 | 0.91 | 0.94 (0.31–1.57) | 1.00 | 242 | 311 | 1.50 | 0.86 (0.69–1.03) | 0.29 | 1.10 (0.74–1.63) | 0.86 |
| **Crohn’s disease** | 6 | 12 | 0.48 | 4.18 (3.20–5.16) | <.01 | 493 | 682 | 1.30 | 1.06 (0.95–1.18) | 0.45 | 3.94 (3.08–5.03) | <.01 |
| **Autoimmune hepatitis** | 0 | 1 |  |  |  | 34 | 54 | 2.28 | 0.70 (0.27–1.13) | 0.45 |  |  |
| **Granulomatosis with polyangiitis** |  |  |  |  |  | 11 | 17 | 3.52 | 0.44 (-0.32–1.20) | 0.80 |  |  |
| **Microscopic polyangiitis** | 0 | 2 |  |  |  | 8 | 18 | 1.71 | 1.31 (0.48–2.15) | 0.58 |  |  |
| **Eosinophilic granulomatosis with polyangiitis** |  |  |  |  |  | 6 | 9 | . | . |  |  |  |
| **Polyarteritis nodosa** |  |  |  |  |  | 6 | 8 | 3.00 | 0.44 (<0.0001–1.50) | 0.74 |  |  |
| **Takayasu’s arteritis** | 0 | 1 |  |  |  | 13 | 10 | 2.00 | 0.38 (<0.0001–1.21) | 0.80 |  |  |
| **Multiple sclerosis** | 0 | 2 |  |  |  | 75 | 98 | 1.93 | 0.68 (0.38–0.98) | 0.27 |  |  |
| **Psoriasis** | 71 | 86 | 1.01 | 1.20 (0.88–1.51) | 0.56 | 2,859 | 3,449 | 1.16 | 1.04 (0.99–1.09) | 0.28 | 1.15 (0.99–1.34) | 0.20 |
| **Type 1 diabetes mellitus** | 41 | 56 | 0.95 | 1.44 (1.03–1.84) | 0.08 | 1,204 | 1,145 | 1.34 | 0.71 (0.63–0.79) | <.01 | 2.02 (1.48–2.75) | <.01 |
| **Hashimoto’s disease** | 189 | 210 | 1.03 | 1.08 (0.88–1.28) | 0.83 | 5,303 | 6,539 | 1.10 | 1.12 (1.08–1.15) | <.01 | 0.97 (0.90–1.03) | 0.55 |
| **Graves’ disease** | 171 | 183 | 0.99 | 1.08 (0.87–1.29) | 0.90 | 5,248 | 6,125 | 1.11 | 1.05 (1.01–1.09) | 0.03 | 1.03 (0.96–1.11) | 0.55 |

COVID-19, coronavirus 2019; neSR, null-effect sequence ratio; aSR, adjusted sequence ratio; CI, confidence interval; BH-adjusted p-value, Benjamini-Hochberg-adjusted p-value
