# Supplementary material for: Association between coronavirus disease 2019 and new-onset autoimmune diseases during the early phase of the pandemic
Source: PLoS One. 2026 May 5;21(5):e0347872. doi: 10.1371/journal.pone.0347872 (PMC13143056; doi:10.1371/journal.pone.0347872)
Supplement: S5 Table — (DOCX) [file pone.0347872.s005.docx]

**S5 Table. Autoimmune disease events within 180 Days in COVID19/non-COVID-19 groups after the index date**

|  | **COVID-19**  **(n=2,678)** | | **Non-COVID-19**  **(n=92,725)** | |
| --- | --- | --- | --- | --- |
|  | **Number of events within 180 days** | **Incidence rate within 180 days** | **Number of events within 180 days** | **Incidence rate within 180 days** |
| **Autoimmune disease** | 1,368 | 0.511 | 48,904 | 0.527 |
| **Autoimmune rheumatic disease^a^** | 639 | 0.239 | 23,789 | 0.257 |
| **Inflammatory bowel disease^b^** | 42 | 0.016 | 1,382 | 0.015 |
| **Autoimmune endocrine disease^c^** | 599 | 0.224 | 19,695 | 0.212 |
| **Systemic lupus erythematosus** | 31 | 0.012 | 1,443 | 0.016 |
| **Systemic sclerosis** | 1 | 0.000 | 115 | 0.001 |
| **Idiopathic inflammatory myopathy** | 3 | 0.001 | 176 | 0.002 |
| **Sjögren diseasee** | 35 | 0.013 | 1,407 | 0.015 |
| **Mixed connective tissue disease** | 1 | 0.000 | 63 | 0.001 |
| **Behcet’s disease** | 9 | 0.003 | 366 | 0.004 |
| **Polymyalgia rheumatica** | 6 | 0.002 | 152 | 0.002 |
| **Rheumatoid arthritis** | 559 | 0.209 | 21,073 | 0.227 |
| **Ankylosing spondylitis** | 20 | 0.007 | 608 | 0.007 |
| **Adult-onset Still’s disease** | 2 | 0.001 | 15 | 0.000 |
| **Ulcerative colitis** | 29 | 0.011 | 969 | 0.010 |
| **Crohn’s disease** | 17 | 0.006 | 457 | 0.005 |
| **Autoimmune hepatitis** | 1 | 0.000 | 78 | 0.001 |
| **Granulomatosis with polyangiitis** | . |  | 24 | 0.000 |
| **Microscopic polyangiitis** | 2 | 0.001 | 23 | 0.000 |
| **Eosinophilic granulomatosis with polyangiitis** | . |  | 12 | 0.000 |
| **Polyarteritis nodosa** | . |  | 13 | 0.000 |
| **Takayasu’s arteritis** | 1 | 0.000 | 16 | 0.000 |
| **Multiple sclerosis** | 4 | 0.001 | 135 | 0.001 |
| **Psoriasis** | 135 | 0.050 | 5,342 | 0.058 |
| **Type 1 diabetes mellitus** | 70 | 0.026 | 1,756 | 0.019 |
| **Hashimoto’s disease** | 292 | 0.109 | 9,919 | 0.107 |
| **Graves’ disease** | 263 | 0.098 | 9,252 | 0.100 |
