# Supplementary material for: Association between coronavirus disease 2019 and new-onset autoimmune diseases during the early phase of the pandemic
Source: PLoS One. 2026 May 5;21(5):e0347872. doi: 10.1371/journal.pone.0347872 (PMC13143056; doi:10.1371/journal.pone.0347872)
Supplement: S6 Table — (B) Sequence symmetry ratio for the diagnosis of autoimmune disease between the severe COVID-19 and non-COVID-19 groups (DOCX) [file pone.0347872.s006.docx]

**S6 Table. (A) Sequence symmetry ratio for the diagnosis of autoimmune disease between the non-severe COVID-19 and non-COVID-19 groups**

| **Diagnosis** | **COVID-19** | | | | | **Non-COVID-19** | | | | | **Difference**  **between log(aSR)** | |
| --- | --- | --- | --- | --- | --- | --- | --- | --- | --- | --- | --- | --- |
|  | **Before** | **After** | **neSR** | **aSR**  **(95% CI)** | **BH-adjusted *p*-value** | **Before** | **After** | **neSR** | **aSR**  **(95% CI)** | **BH-adjusted *p*-value** | **RaSR**  **(95% CI)** | **BH-adjusted *p*-value** |
| **Autoimmune disease** | 1,072 | 1,155 | 1.00 | 1.08 (1.00–1.17) | 0.04 | 43,821 | 48,904 | 1.10 | 1.02 (1.00–1.03) | 0.01 | 1.06 (0.99–1.15) | 0.11 |
| **Autoimmune rheumatic disease** | 520 | 531 | 0.99 | 1.03 (0.91–1.15) | 1.02 | 20,576 | 23,789 | 1.13 | 1.03 (1.01–1.05) | 0.01 | 1.00 (0.97–1.03) | 0.82 |
| **Inflammatory bowel disease^a^** | 28 | 35 | 1.02 | 1.23 (0.73–1.73) | 0.86 | 1,093 | 1,382 | 1.33 | 0.95 (0.87–1.03) | 0.36 | 1.29 (1.14–1.47) | <.01 |
| **Autoimmune endocrine disease^b^** | 451 | 508 | 1.01 | 1.12 (0.99–1.24) | 0.30 | 17,970 | 19,695 | 1.11 | 0.99 (0.97–1.01) | 0.24 | 1.13 (1.02–1.25) | 0.03 |
| **Systemic lupus erythematosus** | 60 | 26 | 0.85 | 0.51 (0.05–0.97) | 0.62 | 1,166 | 1,443 | 1.38 | 0.90 (0.82–0.97) | 0.03 | 0.57 (0.12–2.57) | 0.55 |
| **Systemic sclerosis** | 4 | 1 | 0.00 |  |  | 95 | 115 | 2.03 | 0.60 (0.33–0.87) | 0.06 |  |  |
| **Idiopathic inflammatory myopathy** | 5 | 2 | 0.67 | 0.60 (<0.001–2.24) | 0.91 | 129 | 176 | 1.99 | 0.69 (0.46–0.91) | 0.06 | 0.87 (0.01–59.3) | 0.95 |
| **Sjögren disease** | 23 | 28 | 1.06 | 1.15 (0.60–1.70) | 0.88 | 841 | 1,407 | 1.54 | 1.09 (1.00–1.17) | 0.05 | 1.05 (0.97-1.14) | 0.25 |
| **Mixed connective tissue disease** | 1 | 1 |  |  |  | 71 | 63 | 3.30 | 0.27 (<0.0001–0.61) | 0.61 |  |  |
| **Behcet’s disease** | 8 | 7 | 0.57 | 1.53 (0.52–2.55) | 0.64 | 303 | 366 | 1.98 | 0.61 (0.46–0.76) | <.01 | 2.51 (1.88–3.36) | <.01 |
| **Polymyalgia rheumatica** | 3 | 5 | 1.33 | 1.25 (<0.0001–2.68) | 0.93 | 108 | 152 | 2.97 | 0.47 (0.23–0.72) | 0.03 | 2.64 (0.04–167.9) | 0.70 |
| **Rheumatoid arthritis** | 436 | 471 | 0.99 | 1.09 (0.96–1.22) | 0.51 | 18,430 | 21,073 | 1.13 | 1.01 (0.99–1.03) | 0.43 | 1.08 (1.00–1.17) | 0.09 |
| **Ankylosing spondylitis** | 12 | 16 | 0.97 | 1.38 (0.63–2.13) | 0.55 | 483 | 608 | 1.60 | 0.79 (0.67–0.91) | 0.01 | 1.75 (1.42–2.17) | <.01 |
| **Adult-onset Still’s disease** | 1 | 1 |  |  |  | 25 | 15 | 9.11 | 0.07 (<0.0001–0.71) | 0.28 |  |  |
| **Ulcerative colitis** | 20 | 25 | 0.90 | 1.38 (0.79–1.97) | 0.42 | 747 | 969 | 1.39 | 0.94 (0.84–1.03) | 0.26 | 1.48 (1.17–1.87) | <.01 |
| **Crohn’s disease** | 9 | 13 | 1.22 | 1.18 (0.33–2.03) | 0.85 | 369 | 457 | 1.64 | 0.75 (0.62–0.89) | 0.01 | 1.57 (1.20–2.05) | <.01 |
| **Autoimmune hepatitis** | 1 | 0 |  |  |  | 52 | 78 | 2.92 | 0.51 (0.16–0.86) | 0.16 |  |  |
| **Granulomatosis with polyangiitis** |  |  |  |  |  | 14 | 24 | 4.06 | 0.42 (<0.001–1.08) | 0.72 |  |  |
| **Microscopic polyangiitis** | 0 | 1 |  |  |  | 10 | 23 | 0.92 | 2.50 (1.75–3.24) | <.01 |  |  |
| **Eosinophilic granulomatosis with polyangiitis** |  |  |  |  |  | 8 | 12 | 0.25 | 6.00 (5.11–6.89) | <.01 |  |  |
| **Polyarteritis nodosa** |  |  |  |  |  | 10 | 13 | 5.07 | 0.26 (<0.0001–1.08) | 0.59 |  |  |
| **Takayasu’s arteritis** | 0 | 1 |  |  |  | 15 | 16 | 0.63 | 1.70 (0.99–2.40) | 0.04 |  |  |
| **Multiple sclerosis** | 0 | 4 |  |  |  | 108 | 135 | 2.48 | 0.50 (0.25–0.76) | 0.03 |  |  |
| **Psoriasis** | 93 | 122 | 1.00 | 1.31 (1.04–1.58) | 0.16 | 4,796 | 5,342 | 1.18 | 0.95 (0.91–0.99) | 0.03 | 1.38 (1.11–1.71) | 0.01 |
| **Type 1 diabetes mellitus** | 50 | 50 | 0.92 | 1.08 (0.69–1.47) | 0.89 | 1,823 | 1,756 | 1.44 | 0.67 (0.60–0.73) | <.01 | 1.62 (1.46–1.78) | <.01 |
| **Hashimoto’s disease** | 223 | 253 | 0.99 | 1.15 (0.97–1.33) | 0.59 | 8,656 | 9,919 | 1.11 | 1.03 (1.00–1.06) | 0.07 | 1.11 (0.98–1.27) | 0.14 |
| **Graves’ disease** | 198 | 228 | 1.01 | 1.14 (0.95–1.33) | 0.55 | 8,480 | 9,252 | 1.13 | 0.97 (0.94–1.00) | 0.05 | 1.18 (1.05–1.33) | 0.01 |

COVID-19, coronavirus 2019; neSR, null-effect sequence ratio; aSR, adjusted sequence ratio; CI, confidence interval; BH-adjusted p-value, Benjamini-Hochberg-adjusted p-value

**(B) Sequence symmetry ratio for the diagnosis of autoimmune disease between the severe COVID-19 and non-COVID-19 groups**

| **Diagnosis** | **COVID-19** | | | | | **Non-COVID-19** | | | | | **Difference**  **between log(aSR)** | |
| --- | --- | --- | --- | --- | --- | --- | --- | --- | --- | --- | --- | --- |
|  | **Before** | **After** | **neSR** | **aSR**  **(95% CI)** | **BH-adjusted *p*-value** | **Before** | **After** | **neSR** | **aSR**  **(95% CI)** | **BH-adjusted *p*-value** | **RaSR**  **(95% CI)** | **BH-adjusted *p*-value** |
| **Autoimmune disease** | 238 | 213 | 1.03 | 0.87 (0.69–1.06) | 0.21 | 43,821 | 48,904 | 1.10 | 1.02 (1.00–1.03) | 0.01 | 0.86 (0.73–1.01) | 0.06 |
| **Autoimmune rheumatic disease** | 118 | 108 | 1.03 | 0.89 (0.62–1.15) | 1.30 | 20,576 | 23,789 | 1.13 | 1.03 (1.01–1.05) | 0.01 | 0.86 (0.73–1.02) | 0.12 |
| **Inflammatory bowel disease^a^** | 8 | 7 | 0.92 | 0.95 (<0.0001–1.97) | 0.98 | 1,093 | 1,382 | 1.33 | 0.95 (0.87–1.03) | 0.36 | 1.00 (0.01–71.4) | 1.00 |
| **Autoimmune endocrine disease^b^** | 97 | 91 | 0.92 | 1.02 (0.73–1.31) | 1.34 | 17,970 | 19,695 | 1.11 | 0.99 (0.97–1.01) | 0.24 | 1.03 (1.01–1.06) | 0.02 |
| **Systemic lupus erythematosus** | 11 | 5 | 0.75 | 0.61 (<0.001–1.66) | 1.00 | 1,166 | 1,443 | 1.38 | 0.90 (0.82–0.97) | 0.03 | 0.68 (0.01–52.5) | 1.00 |
| **Systemic sclerosis** | 1 | 0 |  |  |  | 95 | 115 | 2.03 | 0.60 (0.33–0.87) | 0.06 |  |  |
| **Idiopathic inflammatory myopathy** | 0 | 1 |  |  |  | 129 | 176 | 1.99 | 0.69 (0.46–0.91) | 0.06 |  |  |
| **Sjögren disease** | 4 | 7 | 1.14 | 1.53 (0.30–2.76) | 1.00 | 841 | 1,407 | 1.54 | 1.09 (1.00–1.17) | 0.05 | 1.41 (1.25–1.58) | <.01 |
| **Mixed connective tissue disease** | 1 | 0 |  |  |  | 71 | 63 | 3.30 | 0.27 (<0.0001–0.61) | 0.61 |  |  |
| **Behcet’s disease** | 2 | 2 |  |  |  | 303 | 366 | 1.98 | 0.61 (0.46–0.76) | <.01 |  |  |
| **Polymyalgia rheumatica** | 1 | 1 |  |  |  | 108 | 152 | 2.97 | 0.47 (0.23-0.72) | 0.03 |  |  |
| **Rheumatoid arthritis** | 106 | 88 | 1.01 | 0.82 (0.54–1.10) | 1.00 | 18,430 | 21,073 | 1.13 | 1.01 (0.99–1.03) | 0.43 | 0.81 (0.62–1.06) | 0.22 |
| **Ankylosing spondylitis** | 1 | 4 |  |  |  | 483 | 608 | 1.60 | 0.79 (0.67–0.91) | 0.01 |  |  |
| **Adult-onset Still’s disease** | 0 | 1 |  |  |  | 25 | 15 | 9.11 | 0.07 (<0.0001–0.71) | 0.28 |  |  |
| **Ulcerative colitis** | 6 | 4 | 0.80 | 0.83 (<0.0001–2.10) | 0.94 | 747 | 969 | 1.39 | 0.94 (0.84–1.03) | 0.26 | 0.89 (0.01–61.5) | 0.96 |
| **Crohn’s disease** | 2 | 4 | 0.67 | 3.00 (1.30–4.70) | 0.01 | 369 | 457 | 1.64 | 0.75 (0.62–0.89) | 0.01 | 3.97 (2.04–7.74) | <.01 |
| **Autoimmune hepatitis** | 0 | 1 |  |  |  | 52 | 78 | 2.92 | 0.51 (0.16–0.86) | 0.16 |  |  |
| **Granulomatosis with polyangiitis** |  |  |  |  |  | 14 | 24 | 4.06 | 0.42 (<0.001–1.08) | 1.00 |  |  |
| **Microscopic polyangiitis** | 0 | 1 |  |  |  | 10 | 23 | 0.92 | 2.50 (1.75–3.24) | <.01 |  |  |
| **Eosinophilic granulomatosis with polyangiitis** |  |  |  |  |  | 8 | 12 | 0.25 | 6.00 (5.11–6.89) | <.01 |  |  |
| **Polyarteritis nodosa** |  |  |  |  |  | 10 | 13 | 5.07 | 0.26 (<0.0001–1.08) | 0.59 |  |  |
| **Takayasu’s arteritis** |  |  |  |  |  | 15 | 16 | 0.63 | 1.70 (0.99–2.40) | 0.04 |  |  |
| **Multiple sclerosis** |  |  |  |  |  | 108 | 135 | 2.48 | 0.50 (0.25–0.76) | 0.03 |  |  |
| **Psoriasis** | 20 | 13 | 1.02 | 0.64 (<0.0001–1.34) | 0.96 | 4,796 | 5,342 | 1.18 | 0.95 (0.91–0.99) | 0.03 | 0.68 (0.01–58.4) | 0.97 |
| **Type 1 diabetes mellitus** | 18 | 20 | 0.92 | 1.21 (0.57–1.85) | 0.94 | 1,823 | 1,756 | 1.44 | 0.67 (0.60–0.73) | <.01 | 1.81 (1.63–2.01) | <.01 |
| **Hashimoto’s disease** | 44 | 39 | 1.15 | 0.77 (0.34–1.20) | 1.00 | 8,656 | 9,919 | 1.11 | 1.03 (1.00–1.06) | 0.07 | 0.75 (0.48–1.17) | 0.31 |
| **Graves’ disease** | 37 | 35 | 0.86 | 1.10 (0.64–1.56) | 1.00 | 8,480 | 9,252 | 1.13 | 0.97 (0.94–1.00) | 0.05 | 1.14 (1.10–1.17) | <.01 |

COVID-19, coronavirus 2019; neSR, null-effect sequence ratio; aSR, adjusted sequence ratio; CI, confidence interval; BH-adjusted p-value, Benjamini-Hochberg-adjusted p-value
