# Supplementary material for: Association between coronavirus disease 2019 and new-onset autoimmune diseases during the early phase of the pandemic
Source: PLoS One. 2026 May 5;21(5):e0347872. doi: 10.1371/journal.pone.0347872 (PMC13143056; doi:10.1371/journal.pone.0347872)
Supplement: S7 Table — (DOCX) [file pone.0347872.s007.docx]

**S7 Table. Summary of previous studies on the association between COVID-19 and incident autoimmune diseases**

| **Ref** | **Article** | **Study population** | **Study period** | **Follow-up period after index date** | **Comparator** | **Autoimmune diseases** | **Type of statistical analysis** | **Results** |
| --- | --- | --- | --- | --- | --- | --- | --- | --- |
| [3] | Chang et al.  (eClinMed) | - United States of America (USA), patients aged ≥18 years  - TriNetX database  - Retrospective cohort study  - Exclusion of the vaccinated population | 01-01-2020 to 12-31-2021 | From 30 days after the index date until 6 months | Propensity score-matched test-negative control group (1:1) | RA, AS, SLE, DM, SSc, SjD, MCTD, BD, PMR, vasculitis, psoriasis, IBD, celiac disease, and T1DM | Cox-proportional hazards regression | - Higher risk of all autoimmune diseases in the COVID-19 cohort |
| [4] | Tesch et al.  (Clin Rheumatol) | - Germany, all ages  - German routine health care data  - Matched cohort study | 01-01-2020 to 12-31-2020  (follow-up until 06-30-2021) | Between 3 and 15 months after the index date | Age-, sex-, and history of autoimmune disease–matched control group (1:3) | Hashimoto’s thyroiditis, Grave’s disease, psoriasis, RA, SjD, T1DM, UC, CD, PMR, MS, sarcoidosis, AS, celiac disease, alopecia areata, vitiligo, ITP, SLE, arteritis temporalis, bullous pemphigoid, MG, GPA, PBC, AIH, SSc, AIHA, SSc, BD, and GBS | Poisson regression | - 42.63% higher likelihood of acquiring autoimmunity  - Greater risk for incident autoimmune disease in patients with more severe COVID-19 |
| [5] | Peng et al.  (eClinMed) | - Hong Kong, patients aged ≥18 years  - Territory-wide electronic medical records  - Population-based cohort study  - Inclusion of the vaccinated population | 04-01-2020 to 11-15-2022 | From the index date to death or diagnosis of autoimmune disease  (sensitivity analysis: from 28 days after the index date to death or diagnosis) | Age- and sex-matched control group | IBD, pernicious anemia, SpA, RA, DM/PM, other autoimmune arthritis, psoriasis, alopecia areata, pemphigus vulgaris, pemphigoid, vasculitis, Hashimoto’s thyroiditis, Grave’s disease, APS, ITP, GBS, ADEM, transverse myelitis, SLE, SjD, SSc, and MS | Inverse probability of treatment weighting (IPTW)-adjusted Cox proportional hazards regression | - Increased risk of pernicious anemia, SpA, RA, other autoimmune arthritis, psoriasis, pemphigoid, Grave’s disease, APS, ITP, MS, and vasculitis in the COVID-19 group  - Lower risk of pemphigoid, Graves’ disease, APL, ITP, SLE, and other autoimmune arthritis after completion of low doses of the COVID-19 vaccine |
| [6] | Lim et al.  (JAMA Network open) | - South Korea  - Population-based data from the KDCA COVID-19 NHIS registry  - Inclusion of the vaccinated population | 10-08-2020 to 12-31-2021 | From the index date to death or diagnosis of autoimmune disease or 12-31-2021 (sensitivity analysis: from the index date to 60 days in the survived population) | Birth year- and sex-matched control group | Alopecia areata, alopecia totalis, psoriasis, vitiligo, ANCA-associated vasculitis, BD, CD, UC, RA, AOSD, PM, SLE, SSc, SjD, AS, and sarcoidosis | Multivariable Cox proportional hazards regression | - Increased risk of alopecia areata, alopecia totalis, ANCA-associated vasculitis, CD, and sarcoidosis in the COVID-19 group |
| [7] | Hileman et al.  (Front Immunol) | - USA, patients aged ≥18 years  - TriNetX database  - Retrospective and population-based cohort study | 01-01-2020 to 03-03-2023 | From 1 month after the index date until 1 year | Age-, sex-, and propensity score–matched control group | Cutaneous vasculitis, PAN, hypersensitivity angiitis, T1DM, MCTD, UC, psoriasis, autoimmune thyroiditis, CNS arteritis, reactive arthritis, ANCA-associated vasculitis, celiac disease, PMR, sarcoidosis, SjD, IIM, RA, SpA, AIH, SSc, AOSD, Graves’ disease, SLE, and CD | Descriptive statistics and risk ratio estimation | - Increased risk of cutaneous vasculitis, PAN, hypersensitivity angiitis, T1DM, MCTD, UC, psoriasis, and autoimmune thyroiditis in the COVID-19 group  - Lower risk after infection with Omicron variants |
| [8] | Kim et al  (Ann Intern Med) | - South Korea and Japan,  Patients aged ≥20 years  - KDCA COVID-19 NHIS database and Japanese claims data  - Binational, longitudinal, propensity-matched cohort study  - Inclusion of vaccinated population in South Korea data | 01-01-2020 to 12-31-2021 | From 30 days after the index date to death or 12-31-2021 | Propensity score–matched control group  and influenza virus group | Autoimmune inflammatory rheumatic disease (AIRD), inflammatory arthritis (RA, psoriatic arthritis, and SpA), and connective tissue disease (SLE, SjD, SSc, PMR, MCTD, DM/PM, PAN, and vasculitis) | Cox proportional hazards regression | - Increased risk of incident AIRD in the COVID-19 group compared with that in the control and influenza virus groups  - Higher risk of incident AIRD in patients with more severe COVID-19 |
| Present study | Park et al. | - South Korea, all ages  - Population-based data from KCDA COVID-19 NHIS registry  - Sequence symmetry analysis | 10-01-2020-to 06-30-2021  (follow-up for at least 6 months) | From 14 days after the index date to 6 months | Age- and sex-matched control group (1:10) | SLE, SSc, IIM, SjD, MCTD, BD, PMR, RA, AS, AOSD, UC, CD, AIH, GPA, EGPA, PAN, TA, MS, psoriasis, T1DMm Hashimoto’s thyroiditis, and Graves’ disease | Sequence symmetry analysis | - Increased risk of BD, AS, UC, CD, psoriasis, T1DM, and Graves’ disease in the COVID-19 cohort |

KCDA, Korea Centers for Disease Control and Prevention Agency; COVID-19, coronavirus disease 2019; NHIS, National Health Insurance Service; SLE, systemic lupus erythematosus; SSc, systemic sclerosis; MS, multiple sclerosis; IIM, idiopathic inflammatory myopathy; SjD, Sjögren disease; MCTD, mixed connective tissue disease; BD, Behcet’s disease; PMR, polymyalgia rheumatica; RA, rheumatoid arthritis; AS, ankylosing spondylitis; AOSD, adult-onset Still’s disease; UC, ulcerative colitis; CD, Crohn’s disease; GPA, granulomatosis with polyangiitis; MPA, microscopic polyangiitis; EGPA, eosinophilic GPA; PAN, polyarteritis nodosa; TA, Takayasu’s arteritis; T1DM, type 1 diabetes mellitus; IBD, inflammatory bowel disease; SpA, spondyloarthropathy; DM, dermatomyositis; PM, polymyositis; AIH, autoimmune hepatitis; APS, antiphospholipid syndrome; GBS, Guillain-Barré syndrome)
